# Barriers to successful recruitment of parents of overweight children for an obesity prevention intervention: a qualitative study among youth health care professionals

**DOI:** 10.1186/1471-2296-13-37

**Published:** 2012-05-16

**Authors:** Sanne MPL Gerards, Pieter C Dagnelie, Maria WJ Jansen, Nanne K De Vries, Stef PJ Kremers

**Affiliations:** 1 Department of Health Promotion, and NUTRIM School for Nutrition, Toxicology and Metabolism, Maastricht University, Maastricht, The Netherlands; 2 Department of Epidemiology, Maastricht University, Maastricht, The Netherlands; 3 School of Public Health and Primary Care (Caphri), Maastricht University, Maastricht, The Netherlands; 4 South Limburg Municipal Health Services, Geleen, The Netherlands

## Abstract

**Background:**

The recruitment of participants for childhood overweight and obesity prevention interventions can be challenging. The goal of this study was to identify barriers that Dutch youth health care (YHC) professionals perceive when referring parents of overweight children to an obesity prevention intervention.

**Methods:**

Sixteen YHC professionals (nurses, physicians and management staff) from eleven child health clinics participated in semi-structured interviews. An intervention implementation model was used as the framework for conducting, analyzing and interpreting the interviews.

**Results:**

All YHC professionals were concerned about childhood obesity and perceived prevention of overweight and obesity as an important task of the YHC organization. In terms of frequency and perceived impact, the most important impeding factors for referring parents of overweight children to an intervention were denial of the overweight problem by parents and their resistance towards discussing weight issues. A few YHC professionals indicated that their communication skills in discussing weight issues could be improved, and some professionals mentioned that they had low self-efficacy in raising this topic.

**Conclusions:**

We consider it important that YHC professionals receive more training to increase their self-efficacy and skills in motivating parents of overweight children to participate in obesity prevention interventions. Furthermore, parental awareness towards their child’s overweight should be addressed in future studies.

## Background

Childhood overweight and obesity are growing problems worldwide [1-3]. A key approach to preventing these problems and their adverse short- and long-term health consequences [4,5] is the development and evaluation of practice- and theory-based health promotion interventions [6]. Efficacy studies have shown that childhood obesity treatment and prevention programmes can be effective [7-9]. The public health impact of these interventions, however, strongly depends on the proportion of the target group that is exposed to the intervention [9,10].

One of the challenging aspects of implementing interventions to prevent or treat childhood overweight and obesity is the recruitment of participants [11,12], which is often considerably more difficult than expected [11]. Recruitment may be particularly challenging when one or more of the other family members have to be engaged in the intervention programme. But even if only the children and not their parents participate in the intervention, the parents may be the largest obstacle to recruiting children for participation in weight-management programmes [12].

A general distinction can be made between active and passive recruitment methods [13]. Active methods (or interpersonal channels) are methods in which researchers identify and approach potential participants (e.g., by phone, by mail or in person), whereas in passive methods subjects have to identify themselves as potential participants after exposure to, for instance, mass media channels, flyers and posters. A study comparing active and passive recruitment found that active recruitment, i.e. paediatrician referral and direct mail, produced the highest inclusion rate [14], but a variety of obstacles to the active recruitment of children and their parents have been reported [15].

In the Netherlands, youth health care (YHC) is a unique system of preventive health care for all children aged 0–19 years [16]. YHC professionals (physicians and nurses) systematically monitor the physical, psychological, social and cognitive health of children and advise parents and children on achieving a healthy development for the child in these respects. YHC professionals also signal possible health problems such as growth impairment, depression, aggression and overweight. If necessary, the YHC organization offers effective support or refers children to other health care facilities [16]. The YHC service is offered by the government free of charge, and participation is voluntary. Annually, more than 90% of the 0–4 year old children are reached (e.g., in 2009, almost all 0-year-old children were reached and approximately 80% of the 4-year-old children) [17]. This high level of reach makes the YHC service, in theory, an optimal setting to actively recruit children and their parents for health promotion programmes. YHC professionals are a potential gateway to childhood obesity interventions, in line with systems that currently operate in Dutch primary health care with respect to adult obesity treatments. Although parents may constitute an obstacle in terms of recruitment, YHC professionals could be expected to be optimally equipped to enroll participants for childhood obesity programs. Nevertheless, recruitment problems in obesity prevention interventions have also been reported in the Dutch YHC setting [18]. However, no qualitative studies exist that aim to gain insights into the reasons of the recruitment problems in the Netherlands.

The goal of the present study was to identify barriers that Dutch YHC professionals perceive when referring parents of overweight children to an obesity prevention intervention. The study used a qualitative theory-based research design that applied semi-structured interviews.

## Methods

Before the research methodology of the present study is outlined, we will first provide relevant information regarding the childhood obesity intervention and the referral procedure.

### Childhood obesity intervention

The present study is part of the pilot phase of a randomized controlled trial, in which the effectiveness will be tested of a 14-week parent-focused group intervention program. The aim of the intervention is to improve parenting skills and parenting practices related to child’s nutrition and physical activity behaviours. The pilot intervention was aimed at parents of overweight children aged 4 years.

### Referral procedure

**Figure 1:**
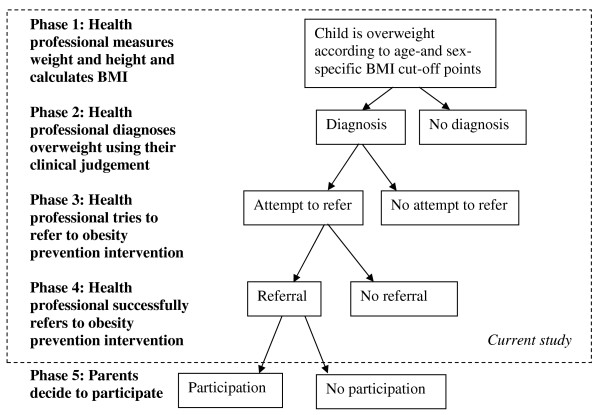
Recruitment procedure.

The referral procedure for the pilot implementation of the obesity prevention intervention consisted of five phases, which are depicted in Figure 1. Phase 1 and 2 are part of the current standard procedures of YHC in the Netherlands [19]. At age 3 years and 9 months, children were systematically invited for a preventive visit to child health clinics, where their growth and health behaviours were assessed by a YHC physician. If a child was labelled ‘overweight, not obese’, according to the sex-and age-specific cut-off points for overweight and obesity based on Cole et al. 2000 [5] (phase 1), and according to the physicians’ clinical judgment (based on their experience, expertise and the course of the weight pattern over time) (phase 2), YHC physicians were asked to refer parents to an intervention programme aimed at the prevention of excessive weight gain in 4-year-old overweight children (phases 3 and 4). Parents were approached for participation in the intervention by a member of our study team: parents made their own decision to participate or not (phase 5). In the current study, we tried to identify factors impeding successful implementation of the referral strategy used by the YHC professionals (phases 1–4).

### Implementation of referral procedure

As part of the implementation of the referral procedure, a dissemination strategy was developed in order to optimally communicate the procedure with the YHC professionals. In a plenary information session, YHC professionals received information on the intervention and what was expected from them. They also received a written protocol with the guidelines. To ensure that they would not forget to refer eligible parents of overweight children to the intervention, reminders were sent in the form of monthly emails and newsletters. The health professionals were also given an email address and telephone number of the research team, whom they could consult if they had any questions about the procedure. This recruitment procedure was implemented for approximately six months in 14 child health clinics run by two different health organizations in the southern part of the Netherlands (South Limburg). Twenty-five YHC physicians were asked to refer children. Based on YHC records and national overweight prevalence rate in the age group (14%), an approximate amount of 230 children were eligible for participation in the geographical area during the recruitment period. However, at the end of the recruitment period, the number of referrals proved to be approximately 10% of the eligible children, while we expected this referral rate to be at least double this percentage [18].

### Semi-structured interviews

Semi-structured interviews were conducted as part of an embedded mixed method design (quantitative results are part of an ongoing RCT). The interviews were held with a fairly open framework which allowed focused, conversational, two-way communication. After the six-month implementation period, we asked YHC physicians from the participating child health clinics to participate in semi-structured interviews to identify the reasons for the disappointing number of referrals. To gain a broader insight into potential barriers of recruitment we also invited YHC nurses and management staff members. YHC professionals could register for the interviews when they were willing to participate. All YHC professionals working in the 14 child health clinics were eligible to participate in the interviews. Sixteen YHC professionals participated (response rate 22%). The interviews were held at the offices of the health professionals in March and April 2010, by the first author of this manuscript, S.G. The current study was part of a larger project which was approved by the ethical committee of the Maastricht University Medical Centre (trial number 10-3-052). All of the participants gave permission for the interviews to be recorded on audiotape. The interviews lasted an average of 20 minutes. All questions were open-ended and concerned a range of topics, including prevention of childhood obesity, appraisal of the intervention and barriers and facilitators to the recruitment of children via YHC. Some example questions are: ‘How did you try to refer overweight children to the intervention?’,’ What were your experiences in referring children?’ ‘Did you experience any barriers or stimulating factors in referring children?’.

### Research model

**Figure 2:**
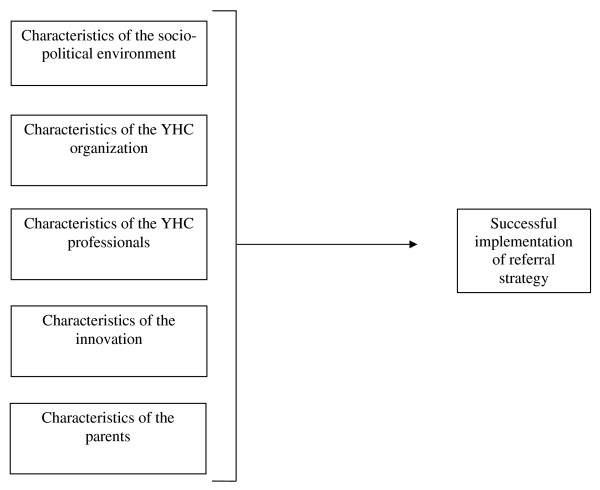
Research model, based on Fleuren et al. (2004) [20].

The research model for the study was based on an implementation theory developed by Fleuren et al. [20]. This theory distinguishes five categories of factors which determine the implementation rate of innovations (see Figure 2): (1) characteristics of the socio-political environment or the context (e.g., norms and values in society);(2) characteristics of the implementing organization, in this case the YHC organization (e.g., tasks, training and cooperation);(3) characteristics of the implementers, i.e. the YHC professionals (e.g., attitude, self-efficacy, skills, remembering to refer);(4) characteristics of the innovation (e.g., relative advantage, observability (degree to which the results of an innovation are visible [21]), relevance to the client and frequency of the innovation) and (5) characteristics of the participants, i.e. the parents (e.g., awareness, perceived severity of the problem, resistance, motivation, perceived responsibility, willingness to cooperate and parental discomfort about the intervention). The research model was used as a framework for conducting, analyzing and interpreting the interviews.

### Data analysis

After the interviews had been transcribed, they were coded by two independent reviewers using NVivo 2.0 software. Both reviewers used a coding list which had been drafted before the coding procedure. The framework for analysis of the transcripts was related to the topics in our research model (Figure 2). If no suitable code was available, the reviewers together determined the relevance of adding an extra code. After they had both coded the interviews, the reviewers compared their codings, and disagreements were solved in a consensus meeting with a third reviewer.

## Results

### YHC professionals

Interviews were conducted with two management staff members, eight physicians and six nurses. Demographic characteristics of the health professionals are depicted in Table 1. All respondents, except one, were female, and their average age was 46 (range 28–61) years. The respondents worked in eleven different child health clinics, almost all were part-timers, and they had an average of 16 years of work experience (range 3–28 years).

**Table 1:** Characteristics of the YHC professionals

**Practice**	**(N = 11)**
Socio-economic status population:	
Low	3
Moderate	6
High	2
**Participants**	**(N = 16)**
Professional Group:	
Management	2
Physician	8
Nurse	6
Gender:	
Female	15
Male	1
Age:	
< 39 years	5
40 – 49 years	4
> 50 years	7
Years since qualification:	
< 10 years	4
– 19 years	7
20 – 39 years	5
Appointment:	
Full-time	1
Part-time (< 0.5 fte)	10
Part-time (≥ 0.5 fte)	5

### Characteristics of the socio-political context

With regard to the socio-political context, various societal norms and values were mentioned as hampering the recruitment procedure. Two respondents indicated that norms are changing, so that overweight children are more and more regarded as having a normal weight (see Table 2 for citations). Another important view in society is the idea that participation in child overweight programmes is still unusual. One professional also mentioned that people are not aware of the severity of overweight as a problem.

**Table 2:** Stimulating and impeding factors in the recruitment of overweight children

**Factors**	**Quotations to illustrate the identified factors**
*Socio-political context*	
Norms and values in society	
Weight-related	(–) ‘The norm about what is normal weight is changing.
	Children who have a healthy weight are now regarded as too lean’
	(–) ‘Parents don’t recognize their child being overweight, because there’s an increase in the number of fat children’
Participation in programmes	(–) ‘It should be regarded as normal that parents participate in such a programme’
Severity of overweight problem	(–) ‘Overweight should get more attention in the media to make people aware that it’s an important health problem’
*YHC organization*	
Task of YHC	(+) ‘Yes, absolutely! I think that no one else will call the parents to account for their child’s overweight’
	(+) ‘Youth health care is the only place where you can find all those young children. There’s no other place to reach all children’
	(+) ‘We are partly responsible, but we’re not the only ones who are responsible. Primary schools have responsibilities as well, as they see what the children eat’
Training	(–) ‘Extra skills training for professionals on the prevention of childhood overweight is needed in YHC’
Time	(–) ‘Of course you make time for it, but there’s not much time to discuss it, and a lot of other things have to be discussed as well during a consultation’
Resources	(–) ‘I think we don’t have enough resources’
Cooperation	
Within the organization	(–) ‘Nurses should be more closely involved in the recruitment of overweight children’
	(–) ‘Unfortunately, we nurses didn’t receive any information’
Between organizations	(–) ‘We think that we should first try to get all relevant stakeholders to agree’
	(+) ‘The more consistent people from different organizations are in their message to parents, the higher the chance that we reach them’
	(+) ‘The municipal health service is also involved in prevention of childhood obesity.’
	(+) ‘It would be better if more organizations were involved. I mean general practitioners, schools, day-care centres’
*The YHC professional*	
Attitude	
Programme - specific outcome beliefs	(+) ‘I think it’s a very positive programme, because overweight is mainly an educational problem’
	(+) ‘Yes, I think it’s a nice programme, although it’s intensive. It takes a lot of time for the parents’
Target group - specific outcome beliefs	(–) ‘I don’t know. I wonder whether parents see the need for it’
	‘I think a particular group does, but I don’t think that all parents see the relevance’
	(+) ‘We do notice an increased need for parenting support’
	(–) ‘I think we all thought: how can we motivate these parents? from the very beginning’
	(–) ‘A lot of people feel uncomfortable when they hear their child is overweight. And that makes it hard for us’
	(–) ‘They’re not interested. They have no time for it’
	(–) ‘I think that one out of thirty people consciously want to change something’
	(–) ‘You notice that parents are very unresponsive’
Perceived responsibility	(+) ‘I feel responsible and want to discuss it with parents’
	(+) ‘It’s a growing problem’
Perceived severity of problem	(–) ‘Obesity is just one of the areas of special interest. I can’t say it’s more important than other areas; it’s just one of them, although I take it very seriously!’
	(–) ‘To be honest, I think that a lot of children between S1 and S2 are actually not too fat’
	(–) ‘My experience is that the majority of the overweight children are just above the norm. When you look at that child, using your clinical judgement, I think those children are not overweight’
Need for prevention	(+) ‘Prevention of childhood obesity is really important, especially to prevent long-term risks’
	(–) ‘I think that prevention of psychosocial problems is more important, but by that I mean severe problems like neglect’
Self – efficacy	(–) ‘I think it’s a complicated problem’
	(–) ´Sometimes parents were very critical and started asking me a lot of questions, which I couldn´t answer. I felt uncomfortable´
Skills	(–) ‘We’re not able to communicate the impact of the problem to the parents. We need more practice in communication skills’
	(–) ‘I think we don’t have enough expertise about prevention of childhood obesity’
Forgetting	(–) ‘I have to admit, I had forgotten it after a while’
*The innovation*	
Relative advantage	‘I think that the current protocol and the intervention can complement each other’
	(+) ‘An advantage of the intervention is that professionals who are experienced in childhood obesity give the parents advice’
	(–) ‘I think that we’re already quite effective in our own approach’
	(+) ‘I think it’s a very positive programme, because overweight is mainly an educational problem’
Observability	(–) ‘I would prefer to get more timely feedback on which parents participated and which ones didn’t’
Relevance for the client	(+) ‘I think that the programme is very useful for parents. Childhood overweight is a problem which is closely related to parenting’
Low frequency of use innovation	(–) ‘No, I didn’t see any children who were eligible for participation’
*The parents*	
Awareness of child’s overweight	(–) ‘Parents are not aware of their child’s overweight’
Perceived severity of child’s overweight	(–) ‘Parents often don’t see their child’s overweight as a problem’
Resistance in discussing weight issues with parents	(–) ‘You clearly notice that parents are unresponsive’
	(–) ‘You notice that when you mention the word ‘overweight’ to parents, you immediately perceive resistance’
	(–) ´Sometimes parents become angry when you continue to discuss overweight’
Motivation to change behaviour	(–) ‘Parents are just not motivated to change their behaviour’
	(–) ‘It’s difficult to approach parents for overweight prevention; they just don’t see the long-term advantages’
Perceived responsibility	(–) ‘Parents don’t admit that they are themselves responsible for the weight of their child’
Willingness to cooperate in the intervention	(–) ‘Some parents want to reduce their child’s overweight themselves’
	(–) ‘They mention that they are closely watching their child’s weight themselves and that they already know what to do about it’
	(–) ‘A lot of parents don’t have time for it. Or they don’t want to make time for it’
Parent’s discomfort about the intervention	(–) ‘Parents think that it’s going too far to participate in an intervention’

### Characteristics of the YHC organization

All respondents considered prevention of childhood obesity to be an important task of the YHC organization, although some professionals commented that other stakeholders had responsibilities as well. General practitioners, schools, day-care centres and municipal health services were mentioned as other potentially relevant stakeholders.

Few YHC professionals reported a need for extra skills training on the management of childhood obesity. Some of them indicated that they had insufficient time during the consultations to refer children to the intervention, or that not enough resources were available. As regards cooperation, some interviewees indicated that it would be important to involve nurses in the recruitment of children as well, because they often know more about the children’s background.

### Characteristics of the YHC professionals

A number of socio-cognitive factors of the YHC professionals were identified as potentially stimulating or impeding factors in the recruitment of overweight children. Respondents mentioned both outcome beliefs about the programme as well as beliefs about the target group as influencing the recruitment of overweight children. The majority of the respondents were positive about the intervention; they thought the intervention was useful, although some physicians mentioned that it was intensive and time-consuming. In general, respondents were less positive about whether they expected the target group to cooperate in the innovation. When professionals expect that the target group does not want an intervention or does not see the need for it, they may be less likely to refer. Most of the interviewees perceived the prevention of childhood obesity to be among their responsibilities. The interviewees reported mixed beliefs regarding the perceived severity of the problem. Although more than half of the respondents perceived childhood overweight and obesity as a serious problem, some mentioned that children who were overweight according to the guidelines were not always overweight according to their clinical judgement. All respondents considered prevention of overweight to be important.

A few respondents indicated that their skills (e.g. communication skills) in the prevention of childhood obesity could be improved, and some professionals mentioned that they had low self-efficacy. Both a lack of communication skills and low self-efficacy were expected to hamper the recruitment of parents of overweight children. A final barrier was that some professionals forgot to implement the recruitment procedure.

### Characteristics of the innovation

Various characteristics of the intervention were mentioned by the interviewees as influencing the recruitment of parents of overweight children. Respondents’ views about the relative advantage of the intervention were mixed. Although some mentioned clear advantages, others thought another new intervention to manage childhood obesity was superfluous. Regarding the observability of the recruitment procedure, some respondents mentioned that it was not always clear to them whether the parents they referred actually participated in the intervention. Nevertheless, almost all respondents were convinced of the relevance of an obesity prevention programme for parents. One third of the respondents indicated that they had had no eligible participants in their clinic during the recruitment period, and they had therefore not been parents able to refer of overweight children.

### Characteristics of the parents

The majority of the professionals indicated that parents seemed to be unaware of their children’s overweight, or unaware of the health consequences (low perceived severity). Several respondents indicated that they had encountered resistance on the part of the parents when they tried to discuss their children’s weight or overweight with them. Some respondents had even found that parents became angry. Almost all YHC professionals also observed a lack of motivation among parents to change behaviour, sometimes because people did not recognize the advantages of behavioural change. Some respondents mentioned that some parents had low perceived responsibility; they appeared not to understand that they were partly responsible for their child’s weight. Others indicated that some parents said they preferred to try and reduce their child’s weight themselves. Some parents had also said that they did not have enough time, or had argued that they did not see the need to participate in an intervention.

## Discussion

The current study investigated the views of Dutch youth health care (YHC) professionals regarding barriers to referring parents of overweight children to an obesity prevention intervention. Although the Dutch YHC system appears an excellent opportunity to identify and refer overweight children, and all interviewed YHC professionals were concerned about childhood obesity and perceived prevention of overweight and obesity as an important task of the YHC organization, a range of barriers impeded the optimal referral. In terms of frequency and perceived impact, the most important aspects hampering the referral procedure were reported to be factors relating to the parents of the overweight children. In particular, denial of the overweight problem and resistance towards discussing weight issues were often mentioned by respondents as impeding factors. Some interviewees reported themselves to be unable to motivate parents to participate in an obesity intervention. Their (self-perceived) insufficient skills and low self-efficacy towards motivating parents are likely to hamper recruitment. Other relevant factors hindering the recruitment process were related to the organization (e.g., lack of time, lack of resources, lack of skills training), societal norms related to weight status, and societal norms towards participation in child overweight programs. In fact, the changing norm in society towards ‘normal weight’ may also have been visible in the observation that some of the children that were referred were actually obese. The acceptance of early interventions in society is expected to rise when evidence-based interventions become more prevalent and easily accessible. This may involve that obesity prevention interventions are not presented as part of the primary health care domain, but as part of the public health domain. Recruitment through public settings such as schools and communities may then complement YHC referral strategies.

No previous study has identified barriers that Dutch YHC professionals perceive when referring parents of overweight children to an obesity prevention intervention. Earlier studies in other health care contexts did however also report barriers to managing childhood obesity as perceived by physicians or nurses. In parallel to our results, those studies also identified perceived difficulties among YHC professionals in discussing weight issues with parents [12,22]. Barriers were reported especially when physicians experienced personal weight challenges [23], or in situations when children lacked motivation [18,24,25], and when there was a lack of family involvement or motivation [18,23-25], a lack of support services [23,25,26] or a lack of time [23,26-29]. Our study showed that in general, YHC professionals do acknowledge the relevance of early prevention. Some of them indeed did not see the advantage of our parent-focused group intervention program, which may also be a reason for a lack of referrals. YHC professionals do acknowledge their professional responsibility to raise the issue of excessive weight gain in children. However, they appear uncomfortable and unequipped to do this. Previous research among physicians has also found that low perceived skills [23,25,28], low self-efficacy [22,23,26,30], and low priority for the overweight problem [31], hamper the recruitment of overweight children for prevention and treatment programmes.

Poor detection of overweight [15], probably due to low use of BMI-for-age [32,33], has also been indicated to be a problem in referral strategies. Indeed, it is questionable whether an obesity prevention intervention should be advocated for a child identified as just in the overweight range based on a single assessment. It would be even more challenging to raise the topic of overweight with parents of children that have always been in the healthy weight range and are just in the overweight range at one point in time. Dutch guidelines, however, do not incorporate only one measurement in the classification of overweight in children. YHC physicians take the course of children’s weight development over time into account in determining whether a child is overweight (i.e. ‘clinical judgment’). This clinical judgement is a vague criterion which may make it relatively difficult to refer parents. This could be an interesting topic for future clinical studies, also in order to provide feedback to YHC professionals with the goal to optimize their clinical judgement skills. Some parents, however, appear not to consider their child to be overweight, even though the age- and sex-specific BMI cut-off points indicate they are [34-36]. And parents who do recognize that their child is overweight sometimes lack a perceived need to manage their child’s weight [15,18].

Some strengths and limitations of the present study should be acknowledged. Strengths include the theoretical basis and the use of a qualitative research design, which adds to the richness of the data. We used a research framework that was based on implementation theory to guide the interview structure. The semi-structured qualitative interviews enriched the contents of the broad concepts that were included in the applied research framework. Thus, we ensured that all potentially relevant concepts were addressed in the interviews and we succeeded in getting a grip on the most important beliefs of the interviewed YHC professionals within each concept. All interviews were conducted by the same researcher in order to increase consistency in that data gathering process, and were coded by two independent researchers to increase confirmability (objectivity and neutrality). Limitations of the current study include the selectivity of the sample and the risk of socially desirable answers that is inherent in interviews. Since participation in the interviews was voluntary this may limit the generalisability of the study. We did not have the impression that social desirability was a problem. The professionals appeared to be open in stating their opinions and by admitting their shortcomings and lack of skills. Also, the atmosphere during the interviews was quite confidential. Another limitation of the current study is that we focused exclusively on YHC professionals. YHC professionals could have displayed an external locus of control when they mention that the parents are the main reason for not referring children. On the other hand, some professionals admitted that they have too few skills to communicate effectively with the parents, thereby internally attributing the low referral rates. Studies that focus on the parent perceptions may provide more information on this.

Based on the present results, we consider it important that YHC physicians and nurses receive more training in interview techniques to strengthen their self-efficacy towards discussing weight issues with parents. Motivational Interviewing (MI) may be a fruitful communication strategy in this respect. MI is a client-oriented method based on the use of communication skills to understand individuals’ motivation for change [37]. Studies have shown that MI can be a valuable health behaviour change intervention for children and their parents [38], which can also be used in the treatment of childhood obesity [39,40]. The technique has been successfully applied in health care settings [41]. To increase its effectiveness, the technique should not just be an important intervention component, but should also become a permanent part of professionals’ routine work patterns. Furthermore, a better understanding of parental resistance and denial in relation to child overweight could be gained by future research investigating parents’ views about discussing weight-related issues with health professionals.

## Conclusions

The YHC context and the professionals’ attitude towards prevention of childhood obesity do not appear to be major barriers for referring parents of overweight children to an obesity prevention intervention. By contrast, the professionals particularly perceived factors relating to the parents of the overweight children as impeding optimal referral. This lack of parental awareness towards their child’s overweight should be addressed in future studies. In addition, YHC professionals’ communication skills and self-efficacy in discussing weight issues with parents of overweight children appear insufficient. In addition to efforts to optimize the efficacy of obesity prevention interventions, it will be fruitful to emphasize the importance of increasing the reach of such programmes, for example by training YHC professionals in motivating parents of overweight children to participate in obesity prevention interventions.

## Abbreviations

BMI, Body Mass Index; MI, Motivational Interviewing; YHC, Youth Health Care.

## Competing interests

The contributing authors declare that they have no competing interests with respect to this work.

## Authors’ contributions

SG was the primary investigator in this study and wrote the first draft of the paper. PC, MJ, NdV and SK were involved in revising the manuscript. All authors have read and approved the final manuscript.

## Pre-publication history

The pre-publication history for this paper can be accessed here:

http://www.biomedcentral.com/1471-2296/13/37/prepub
